# Factors influencing postoperative hyperbilirubinemia in valvular heart disease and establishment of a predictive model

**DOI:** 10.3389/fcvm.2025.1611427

**Published:** 2026-01-06

**Authors:** Chenchen Cheng, Haiping Wang, Baoguo Zhou, Zhenqian Lv, Xiaojun Liu, Gang Wang, Yan Qiao

**Affiliations:** Department of Cardiovascular Surgery, Qingdao Cardiovascular Hospital, Qingdao, Shandong, China

**Keywords:** valvular heart disease, hyperbilirubinemia, risk factors, predictive model, VHD

## Abstract

**Background:**

To explore the influencing factors of postoperative hyperbilirubinemia (HB) in patients with valvular heart disease (VHD) and establish a predictive model based on these factors.

**Methods:**

Clinical data of VHD patients who underwent surgical treatment in Qingdao Cardiovascular Hospital from March 2022 to February 2024 were retrospectively collected. The patients were divided into a modeling group (*n* = 215) and a validation group (*n* = 54) in an 8:2 ratio. The modeling group was further divided into an HB group (*n* = 73) and a non-HB group (*n* = 142) based on whether HB occurred within one week after surgery. Multivariable logistic regression analysis was used to analyze the risk factors for HB in VHD patients. Risk prediction nomogram models were established using R3.6.1 software, and receiver operating characteristic (ROC) curves and calibration curves were plotted to evaluate the predictive performance and accuracy of the nomogram models.

**Results:**

The results of multivariable logistic regression analysis showed that the type of surgery (OR = 4.959, 95% CI: 2.592–9.487), preoperative MELD score (OR = 4.332, 95% CI: 2.061–9.105), CPB time (OR = 3.851, 95% CI: 1.591–9.321), aortic cross-clamp time (OR = 3.667, 95% CI: 1.521–8.841), intraoperative total blood transfusion volume (OR = 4.125, 95% CI: 1.982–8.586), and mechanical ventilation time (OR = 4.089, 95% CI: 2.000–8.362) were risk factors for the occurrence of HB in the modeling group (*P* < 0.05). The ROC analysis results showed that the area under the curve (AUC) of the nomogram model for predicting HB in the modeling group and validation group was 0.901 and 0.904, respectively. The calibration curve analysis results showed good consistency between the predicted and actual occurrence of HB in the predictive model, with Hosmer-Lemeshow chi-square statistics of 4.32 and 1.95 and corresponding *P*-values of 0.821 and 0.199.

**Conclusion:**

The type of surgery, preoperative MELD score, CPB time, aortic cross-clamp time, intraoperative total blood transfusion volume, and mechanical ventilation time are risk factors for the occurrence of HB in patients with VHD. The nomogram model constructed based on these risk factors has good predictive value and accuracy.

## 1 Introduction

Valvular heart disease (VHD) represents a significant proportion of cardiovascular conditions necessitating surgical intervention. With the global population aging at an accelerated pace, VHD has become increasingly prevalent, contributing substantially to cardiovascular morbidity and mortality worldwide (1–3). The primary approach for treating VHD is through open-heart valve replacement or repair under cardiopulmonary bypass (CPB), which remains the cornerstone of clinical management (4, 5). Despite advancements in surgical techniques and perioperative care, a growing number of critically ill patients with complex conditions continue to face suboptimal postoperative outcomes, including severe complications that may lead to death (6).

Hyperbilirubinemia (HB) is a common complication after VHD surgery. According to incomplete statistics, the incidence of HB in VHD patients after surgery is as high as 43.3% (7, 8). It is closely associated with poor perioperative prognosis and can progress to acute liver failure, becoming an important cause of in-hospital mortality (9, 10). Consequently, understanding the factors contributing to postoperative HB and establishing effective prevention strategies are crucial for improving patient outcomes. McSweeney et al. found that patients with postoperative HB after cardiac surgery had a prolonged ICU stay of approximately one week, doubled hospitalization time, and significantly increased mortality compared to non-hyperbilirubinemic patients (11). This suggests that clinical attention should be paid to the prevention and treatment of HB in VHD patients with after surgery.

In recent years, with the increasing epidemiological research on postoperative HB in VHD patients, most studies have also analyzed the influencing factors of HB. Currently, most studies focus on analyzing the relationship between laboratory indicators and postoperative HB in VHD patients. However, there is a notable absence of comprehensive studies investigating the multifactorial influences on the occurrence of HB and the lack of predictive models specifically tailored for this context. Therefore, further exploration of the influencing factors of postoperative HB in VHD patients and the construction of a predictive model for HB based on these factors are of great significance for reducing the risk of HB and improving patient prognosis (12, 13).

Given these gaps, this study aims to fill an important void by exploring the risk factors associated with postoperative HB in VHD patients who underwent surgery at Qingdao Cardiovascular Hospital. By retrospectively examining the clinical data of these patients, we divided them into groups based on whether they developed HB within one week after surgery. This division enabled us to identify key risk factors and develop a predictive model using multivariable logistic regression analysis. Furthermore, we constructed a nomogram model to facilitate the early identification of high-risk individuals, thereby enabling more proactive prevention and treatment strategies.

The establishment of such a predictive model not only aids in identifying patients at higher risk of developing HB but also provides valuable insights into the underlying mechanisms driving this condition. Understanding these mechanisms could pave the way for novel therapeutic approaches aimed at mitigating the impact of HB on patient recovery. Additionally, the application of our predictive model in clinical settings could enhance personalized medicine efforts, allowing healthcare providers to tailor interventions based on individual risk profiles. In summary, this study seeks to contribute significantly to the field of cardiac surgery by providing a robust framework for predicting and managing postoperative HB in VHD patients, ultimately aiming to improve overall patient outcomes and reduce the burden on public health systems.

## 2 Materials and methods

### 2.1 Subject selection

This study was a retrospective study that collected clinical data of VHD patients who underwent surgical treatment in Qingdao Cardiovascular Hospital from March 2022 to February 2024. The patients were divided into a modeling group (*n* = 215) and a validation group (*n* = 54) in an 8:2 ratio. Both the modeling group and validation group were further divided into HB groups and non-HB groups based on whether HB occurred within one week after surgery. In the model group, 73 patients were classified into the HB group, and 142 patients were in the non-HB group. In the validation group, 19 patients experienced HB, and 35 patients did not experience HB. Inclusion criteria were as follows: (1) diagnosed with VHD (14) based on diagnostic criteria and confirmed by electrocardiography, echocardiography, and other examinations; (2) age >18 years; (3) scheduled for open-heart valve replacement or repair under direct vision with CPB; (4) complete clinical data. Exclusion criteria were as follows: (1) concurrent major vascular surgery; (2) malignant tumors; (3) patients undergoing interventional procedures; (4) patients with missing clinical data. The grouping criteria referred to previous literature (15), and patients were divided into an HB group [serum total bilirubin (TBIL) > 34.2 μmol/L] and a non-HB group (TBIL≤34.2 μmol/L) based on whether TBIL concentration was above 34.2 μmol/L in any measurement within one week after surgery. This cut-off value was selected based on established clinical criteria for hyperbilirubinemia and aligns with previous studies in cardiac surgery populations (15). This study was approved by the Ethics Committee of Qingdao Cardiovascular Hospital (2024-QXLX-009) and complies with the ethical standards of the Helsinki Declaration. The Ethics Committee has agreed to waive informed consent.

### 2.2 Data collection

Patient perioperative data were collected through the electronic medical record system. (1) Preoperative data included age, gender, medical history, type of surgery, Acute Physiology and Chronic Health Evaluation II (APACHE II) score, Model for End-Stage Liver Disease (MELD) score, cardiac function, and preoperative laboratory indicators (complete blood count, biochemical markers, coagulation indicators, etc.). (2) Intraoperative data included surgery time, cardiopulmonary bypass (CPB) time, aortic cross-clamp time, and total blood transfusion volume. (3) Postoperative data included the use of extracorporeal membrane oxygenation (ECMO) or intra-aortic balloon pump (IABP) and mechanical ventilation time.

### 2.3 Statistical analysis

The collected data were analyzed using SPSS 27.0. All data were tested for normal distribution. Normally distributed continuous variables were presented as mean ± standard deviation. The *t*-test was used for comparisons between two groups, while the *F*-test was used for comparisons among multiple groups. Categorical data were presented as counts or rates, and the chi-square test was used for comparisons. Multiple logistic regression analysis was performed to analyze the risk factors for postoperative HB in VHD patients. The risk prediction nomogram model was established using R 3.6.1 software, and the predictive performance and accuracy of the nomogram model were evaluated by plotting receiver operating characteristic (ROC) curves, decision curve analysis (DCA), and calibration curves. A significance level of *P* < 0.05 was considered statistically significant for differences.

## 3 Results

### 3.1 Changes in postoperative total bilirubin (TBIL) concentration in VHD patients

**Figure 1:**
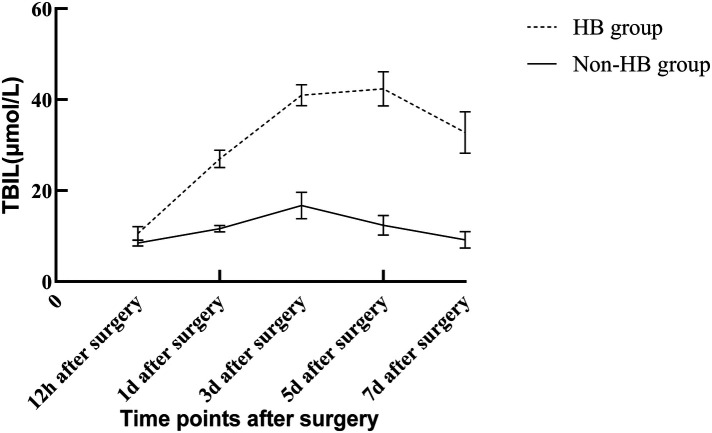
Changes in TBIL concentration at different time points after surgery in the HB and non-HB groups. Data are presented as mean ± standard deviation. HB group (*n* = 73); Non-HB group (*n* = 142). TBIL concentrations were significantly higher in the HB group at all postoperative time points (*P* < 0.001). TBIL, total bilirubin; HB, hyperbilirubinemia.

In the modeling group, 73 cases (33.95%) experienced postoperative HB within the first week, while 142 cases (66.05%) did not. In the HB group, TBIL concentration started to rise within 12 h after surgery, peaked between 3 and 5 days, and gradually decreased. However, even after 7 days, TBIL concentration remained higher than the normal level. In the non-HB group, overall changes in TBIL concentration were relatively small. TBIL concentration was higher within the first 1–3 days after surgery but still within the normal range, and it subsequently decreased. The comparison of TBIL concentrations at different time points after surgery showed statistically significant differences in both groups (*P* < 0.05), as shown in Table 1. The changes in TBIL concentration after surgery in both groups are illustrated in Figure 1.

**Table 1:** Comparison of TBIL concentrations at different time points after surgery in the two groups (μmol/L).

Parameters	*n*	Postoperative 12 h	Postoperative 1 day	Postoperative 3 day	Postoperative 5 day	Postoperative 7 day
HB	73	10.56 ± 1.52	26.97 ± 1.89	40.95 ± 2.33	42.37 ± 3.75	32.79 ± 4.58
Non-HB	142	8.49 ± 0.69	11.63 ± 0.71	16.71 ± 2.89	12.39 ± 2.15	9.15 ± 1.84
*t*		13.728	85.800	62.024	74.472	53.734
*P*		<0.001	<0.001	<0.001	<0.001	<0.001

TBI, total bilirubin; HB, hyperbilirubinemia.

### 3.2 Comparison of patient characteristics data between the HB and non-HB groups

In Table 2, which compares patient characteristics data between the HB (*n* = 73) and non-HB groups (*n* = 142), several factors were found to have significant differences. Specifically, surgery type, preoperative MELD score, and NYHA grade showed statistically significant differences between the two groups (*P* < 0.001). The analysis indicated that patients undergoing aortic valve replacement, having a MELD score ≥12.5, or presenting with higher NYHA grades (III and IV) were more likely to develop postoperative HB. Conversely, demographic factors such as age, gender distribution, smoking history, hypertension, diabetes status, and APACHE II score did not show significant differences between the HB and non-HB groups (*P* > 0.05). These findings highlight the importance of surgical procedure selection, liver function assessment through MELD scores, and cardiac functional evaluation using NYHA grading in predicting the risk of developing postoperative HB in patients with valvular heart disease.

**Table 2:** Comparison of patient characteristics data between the HB and non-HB groups.

Parameters	HB (*n* = 73)	Non-HB (*n* = 142)	χ^2^/*t*	*P*
Age (years)	58.74 ± 6.53	57.69 ± 7.51	1.014	0.312
Gender (Male/Female) (*n*)	34/39	73/69	0.451	0.502
Smoking history (Yes/No) (*n*)	31/42	54/88	0.397	0.529
Hypertension (Yes/No) (*n*)	21/52	36/106	0.289	0.591
Diabetes (Yes/No) (*n*)	8/65	13/129	0.178	0.673
Surgery type (*n*)				
- Aortic valve replacement	19	47	14.902	<0.001
- Mitral valve replacement	30	79		
- Tricuspid valve replacement	24	16		
APACHE II score	17.79 ± 1.82	17.65 ± 1.97	0.506	0.613
MELD score (<12.5/≥12.5) (*n*)	26/47	113/29	40.771	<0.001
NYHA grade				
- II	14	15	44.292	<0.001
- III	19	103		
- IV	40	24		

HB, hyperbilirubinemia; APACHE II, acute physiology and chronic health evaluation II; MELD, model for end-stage liver disease; NYHA, New York Heart Association.

### 3.3 Comparison of laboratory indicators between the HB and non-HB groups

In Table 3, which compares laboratory indicators between the HB and non-HB groups, no significant differences were observed across all evaluated parameters. Specifically, WBC, hemoglobin levels, creatinine concentration, CRP levels, and prothrombin time did not show statistically significant differences between the two groups (*P* > 0.05). These results suggest that common laboratory indicators such as WBC, hemoglobin, creatinine, CRP, and prothrombin time are not significantly associated with the occurrence of postoperative HB in patients undergoing surgical treatment for valvular heart disease.

**Table 3:** Comparison of laboratory indicators between the HB and non-HB groups.

Parameters	HB (*n* = 73)	Non-HB (*n* = 142)	*t*	*P*
White blood cell (×10^9^/L)	7.22 ± 1.25	7.54 ± 1.39	1.653	0.100
Hemoglobin (g/L)	140.6 ± 12.59	143.15 ± 14.67	1.255	0.211
Creatinine (μmol/L)	89.78 ± 9.89	88.62 ± 10.31	0.798	0.426
CRP (mg/L)	8.19 ± 1.65	7.84 ± 1.79	1.394	0.165
Prothrombin time (s)	12.26 ± 1.63	12.48 ± 1.75	0.893	0.373

HB, hyperbilirubinemia; CRP, C-reactive protein.

### 3.4 Comparison of surgical data between the HB and non-HB groups

In Table 4, which compares surgical data between the HB and non-HB groups, several significant differences were identified. Specifically, patients in the HB group were more likely to have longer CPB times (>120 min), prolonged aortic cross-clamp times (≥90 min), and higher intraoperative blood transfusion volumes (≥5U) (*P* < 0.01). Surgery duration did not show a statistically significant difference between the groups (*P* = 0.064). These findings indicate that prolonged CPB and aortic cross-clamp times, as well as increased intraoperative blood transfusion volumes, are associated with an increased risk of postoperative hyperbilirubinemia in patients undergoing surgery for valvular heart disease.

**Table 4:** Comparison of surgical data between the HB and non-HB groups.

Parameters	HB (*n* = 73)	Non-HB (*n* = 142)	χ^2^/*t*	*P*
Surgery time (h)	4.69 ± 0.40	4.48 ± 0.92	1.860	0.064
CPB time (*n*)				
- <60 min	4	16	9.467	0.009
- 60–120 min	55	117		
- >120 min	14	9		
Aortic cross-clamp time (<90/≥90 min) (*n*)	44/29	115/27	10.741	0.001
Intraoperative blood transfusion (<5/≥5 U) (*n*)	24/49	113/29	45.491	<0.001

HB, hyperbilirubinemia; CPB, cardiopulmonary bypass.

### 3.5 Comparison of postoperative recovery data between the HB and non-HB groups

In Table 5, which compares postoperative recovery data between the HB and non-HB groups, significant differences were observed in ECMO or IABP usage and mechanical ventilation duration. The use of ECMO or IABP was significantly higher in the HB group compared to the non-HB group (*P* < 0.001), with 13 patients in the HB group requiring these support measures vs. only 5 in the non-HB group. Additionally, mechanical ventilation duration showed a significant difference between the two groups (*P* < 0.001). In the HB group, none of the patients had zero hours of mechanical ventilation, whereas 31 patients required ventilation for 1–48 h and 42 patients for more than 48 h. In contrast, in the non-HB group, 2 patients did not require mechanical ventilation, 115 patients needed it for 1–48 h, and 25 patients for more than 48 h. These results indicate that patients who develop postoperative hyperbilirubinemia are significantly more likely to require advanced life support measures such as ECMO or IABP and have longer durations of mechanical ventilation.

**Table 5:** Comparison of postoperative recovery data between the HB and non-HB groups.

Parameters	HB (*n* = 73)	Non-HB (*n* = 142)	χ^2^	*P*
ECMO or IABP usage (Yes/No) (*n*)	13/60	5/137	12.831	<0.001
Mechanical ventilation (*n*)				
- 0	0	2	36.232	<0.001
- 1–48h	31	115		
- >48h	42	25		

HB, hyperbilirubinemia; ECMO, extracorporeal membrane oxygenation; IABP, intra-aortic balloon pump.

### 3.6 Multivariable logistic regression analysis of HB incidence in the modeling group

Using the variables with statistically significant differences in the univariate analysis as independent variables and the occurrence of HB in the modeling group as the dependent variable (assigned as non-HB = 0, HB = 1), a multivariable logistic regression analysis was conducted. The results showed that surgical type (OR = 4.959, 95% CI: 2.592–9.487), preoperative MELD score (OR = 4.332, 95% CI: 2.061–9.105), CPB time (OR = 3.851, 95% CI: 1.591–9.321), aortic cross-clamp time (OR = 3.667, 95% CI: 1.521–8.841), total intraoperative blood transfusion volume (OR = 4.125, 95% CI: 1.982–8.586), and mechanical ventilation time (OR = 4.089, 95% CI: 2.000–8.362) were identified as risk factors for HB incidence in the modeling group (*P* < 0.05), as shown in Table 6. These findings indicate that patients undergoing certain types of valve surgeries, those with higher preoperative MELD scores, longer CPB times, extended aortic cross-clamp times, increased intraoperative blood transfusion volumes, and prolonged mechanical ventilation durations are at significantly higher risk for developing postoperative hyperbilirubinemia. Each of these factors independently contributes to the likelihood of HB occurrence, with odds ratios indicating substantial increases in risk.

**Table 6:** Multivariable logistic regression analysis of HB incidence in the modeling group.

Parameters	*β*	SE	Ward χ^2^	*P*	OR	95% CI
Surgical type	1.601	0.331	23.401	<0.001	4.959	2.592–9.487
MELD score	1.466	0.379	14.963	<0.001	4.332	2.061–9.105
CPB time	1.348	0.451	8.938	0.008	3.851	1.591–9.321
Aortic cross-clamp time	1.299	0.449	8.375	0.012	3.667	1.521–8.841
Intraoperative blood transfusion	1.417	0.374	14.356	<0.001	4.125	1.982–8.586
Mechanical ventilation	1.408	0.365	14.887	<0.001	4.089	2.000–8.362

MELD, model for end-stage liver disease; CPB, cardiopulmonary bypass.

### 3.7 Development and evaluation of the risk prediction model

**Figure 2:**
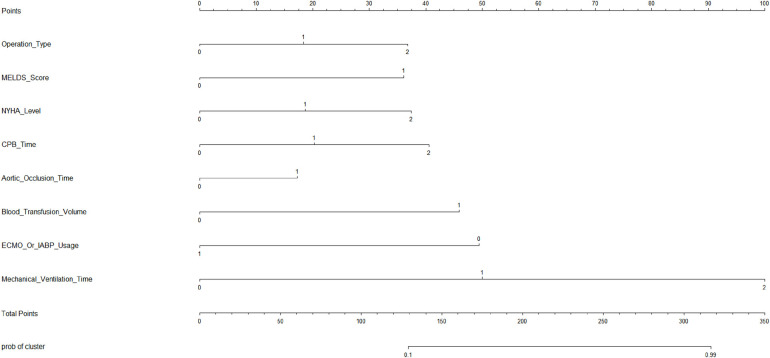
Nomogram of the risk prediction model for HB incidence in VHD patients after surgery. HB, hyperbilirubinemia; VHD, valvular heart disease.

**Figure 3:**
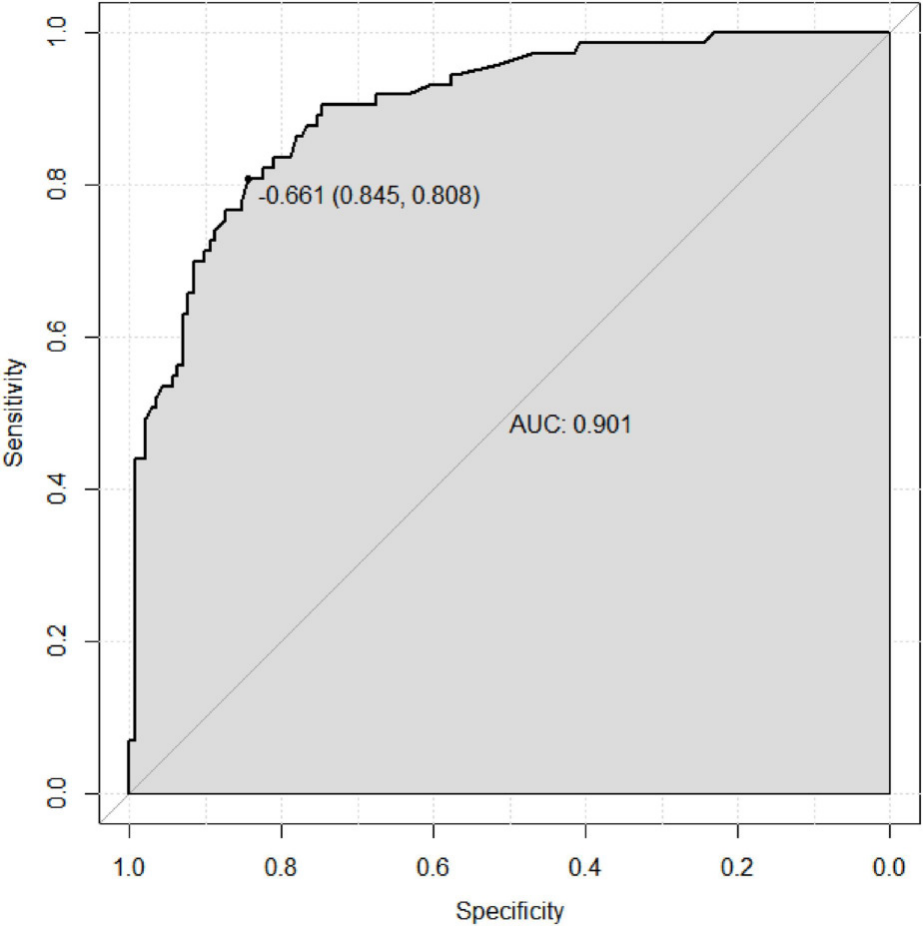
ROC curve of the risk prediction model for HB incidence in the modeling group of VHD patients. ROC, receiver operating characteristic; HB, hyperbilirubinemia; VHD, valvular heart disease.

**Figure 4:**
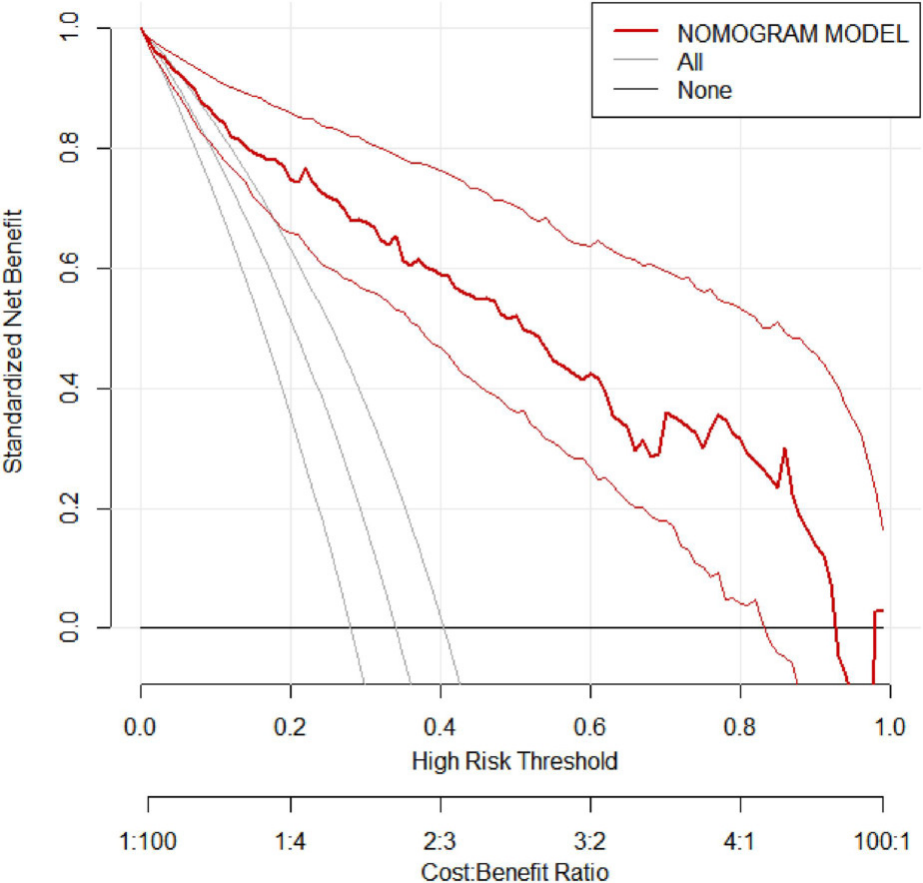
DCA of the risk prediction model for HB incidence in the validation group of VHD patients. DCA, decision curve analysis; HB, hyperbilirubinemia; VHD, valvular heart disease.

**Figure 5:**
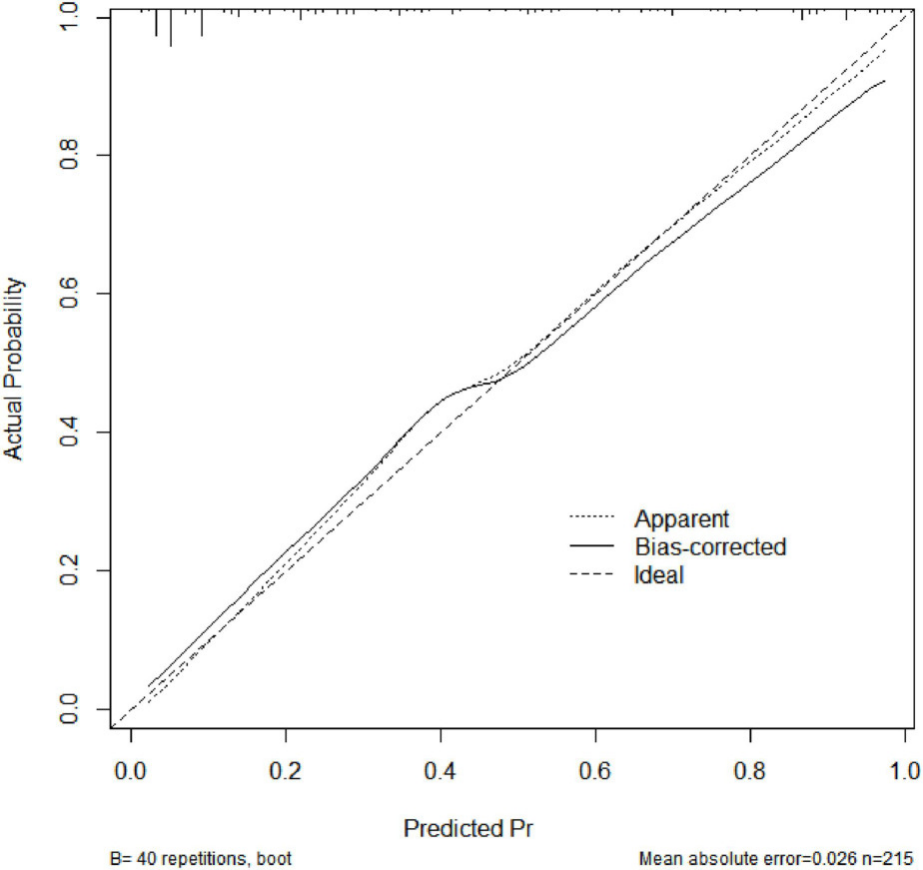
Calibration curve of the risk prediction model in the modeling group of VHD patients. VHD, valvular heart disease.

Based on the results of the multivariable logistic regression analysis, a risk prediction model was developed by incorporating surgical type, preoperative MELD score, CPB time, aortic cross-clamp time, total intraoperative blood transfusion volume, and mechanical ventilation time. The results of the risk prediction model using a nomogram are shown in Figure 2. The ROC analysis results showed that the AUC of the risk prediction model for HB incidence in the modeling group was 0.901, as shown in Figure 3. Based on the risk prediction model, DCA was performed for the identified risk factors for HB. The results showed that using the risk prediction model based on the nomogram yielded higher net benefits in predicting the risk of postoperative HB in VHD patients, as shown in Figure 4. The calibration curve analysis results demonstrated good consistency between the predicted and actual occurrence of HB in the model, with Hosmer-Leme show chi-square statistics of 4.32 and *P*-values of 0.821 for the modeling group, as shown in Figure 5.

### 3.8 External validation

#### 3.8.1 Comparison of perioperative data in the validation group

Table 7 compares perioperative data between the HB (*n* = 19) and non-HB groups (*n* = 35) in the validation cohort, revealing significant differences in surgery type (*P* = 0.004), preoperative MELD score (*P* = 0.001), NYHA grade (*P* = 0.016), CPB time (*P* = 0.006), aortic cross-clamp time (χ^2^ = 10.780, *P* = 0.001), intraoperative blood transfusion volume (*P* < 0.001), ECMO or IABP usage (*P* = 0.030), and mechanical ventilation duration (*P* < 0.001). Patients in the HB group were more likely to undergo tricuspid valve replacement, have higher MELD scores (≥12.5), present with higher NYHA grades (III and IV), experience longer CPB and aortic cross-clamp times, require larger blood transfusions (≥5U), use ECMO or IABP, and need extended mechanical ventilation (>48 h). In contrast, demographic and routine laboratory parameters showed no significant differences. These findings validate the identified risk factors for postoperative hyperbilirubinemia, emphasizing the importance of surgical complexity, liver function, cardiac status, and intensive care needs in predicting and managing this complication.

**Table 7:** Comparison of perioperative data between the HB and non-HB groups in the validation cohort.

Parameters	HB (*n* = 19)	Non-HB (*n* = 35)	χ^2^/*t*	*P*
Age (years)	59.32 ± 7.41	58.20 ± 7.52	0.525	0.600
Gender (Male/Female) (*n*)	8/11	17/18	0.207	0.649
Smoking history (Yes/No) (*n*)	7/12	16/19	0.396	0.529
Hypertension (Yes/No) (*n*)	6/13	10/25	0.053	0.817
Diabetes (Yes/No) (*n*)	4/15	5/30	0.065	0.799
Surgery type (*n*)				
- Aortic valve replacement	3	13	11.239	0.004
- Mitral valve replacement	8	20		
- Tricuspid valve replacement	8	2		
APACHE II score	17.58 ± 1.92	18.00 ± 1.97	0.754	0.454
MELD score (<12.5/≥12.5) (*n*)	6/13	27/8	10.758	0.001
NYHA grade				
- II	4	4	8.227	0.016
- III	6	25		
- IV	9	6		
White blood cell (×10^9^/L)	7.19 ± 1.28	7.63 ± 1.22	1.263	0.212
Hemoglobin (g/L)	139.20 ± 13.84	143.77 ± 13.91	1.155	0.253
Creatinine (μmol/L)	89.97 ± 10.29	87.92 ± 10.46	0.691	0.493
CRP (mg/L)	8.37 ± 2.01	7.83 ± 1.95	0.963	0.34
Prothrombin time (s)	12.22 ± 1.83	12.54 ± 1.44	0.710	0.481
Surgery time (h)	4.52 ± 0.93	4.30 ± 0.97	0.838	0.406
CPB time (*n*)				
- <60 min	1	4	10.330	0.006
- 60–120 min	13	31		
- >120 min	5	0		
Aortic cross-clamp time (<90/≥90 min) (*n*)	9/10	32/3	10.780	0.001
Intraoperative blood transfusion (<5/≥5 U) (*n*)	4/15	32/3	27.447	<0.001
ECMO or IABP usage (Yes/No) (*n*)	5/14	1/34	4.692	0.030
Mechanical ventilation (*n*)				
- 0	0	0	24.137	<0.001
- 1–48 h	6	33		
- >48 h	13	2		

HB, hyperbilirubinemia; APACHE II, acute physiology and chronic health evaluation II; MELD, model for end-stage liver disease; NYHA, New York Heart Association; CRP, C-reactive protein; CPB, cardiopulmonary bypass; ECMO, extracorporeal membrane oxygenation; IABP, intra-aortic balloon pump.

#### 3.8.2 Comparison of perioperative data between the modeling and validation groups

The perioperative clinical data between the modeling group and the validation group were compared, and no statistically significant differences were found (*P* > 0.05), as shown in Table 8. These results indicate that the baseline characteristics and perioperative factors are consistent between the modeling and validation cohorts. This consistency supports the reliability and generalizability of the findings from the modeling group to the validation group, reinforcing the validity of the identified risk factors for postoperative hyperbilirubinemia. The lack of significant differences suggests that the predictive model developed in the modeling cohort can be effectively applied to other similar patient populations.

**Table 8:** Comparison of perioperative data between the modeling and validation cohorts.

Parameters	Modeling (*n* = 215)	Validation (*n* = 54)	χ^2^/*t*	*P*
Age (years)	58.15 ± 7.28	58.62 ± 7.49	0.422	0.674
Gender (Male/Female) (*n*)	107/108	25/29	0.208	0.648
Smoking history (Yes/No) (*n*)	85/130	23/31	0.168	0.682
Hypertension (Yes/No) (*n*)	57/158	16/38	0.212	0.645
Diabetes (Yes/No) (*n*)	21/194	9/45	2.073	0.150
Surgery type (*n*)				
- Aortic valve replacement	66	16	0.028	0.986
- Mitral valve replacement	109	28		
- Tricuspid valve replacement	40	10		
APACHE II score	17.82 ± 1.95	17.67 ± 1.92	0.507	0.613
MELD score (<12.5/≥12.5) (*n*)	139/76	33/21	0.235	0.628
NYHA grade				
- II	29	8	0.117	0.943
- III	122	31		
- IV	64	15		
White blood cell (×10^9^/L)	7.39 ± 1.27	7.45 ± 1.36	0.422	0.674
Hemoglobin (g/L)	141.89 ± 12.72	142.31 ± 14.82	0.306	0.760
Creatinine (μmol/L)	88.97 ± 9.69	88.65 ± 10.25	0.210	0.834
CRP (mg/L)	8.06 ± 1.48	7.95 ± 1.67	0.214	0.830
Prothrombin time (s)	12.22 ± 1.68	12.39 ± 1.74	0.476	0.635
Surgery time (h)	4.54 ± 0.71	4.39 ± 0.88	0.660	0.510
CPB time (*n*)				
- <60 min	20	5	0.098	0.952
- 60–120 min	172	44		
- >120 min	23	5		
Aortic cross-clamp time (<90/≥90 min) (*n*)	159/56	41/13	0.088	0.767
Intraoperative blood transfusion (<5/≥5 U) (*n*)	137/78	36/18	0.163	0.686
ECMO or IABP usage (Yes/No) (*n*)	18/197	6/48	0.399	0.528
Mechanical ventilation (*n*)				
- 0	2	0	0.781	0.677
- 1–48 h	146	39		
- >48 h	67	15		

HB, hyperbilirubinemia; APACHE II, acute physiology and chronic health evaluation II; MELD, model for end-stage liver disease; NYHA, New York Heart Association; CRP, C-reactive protein; CPB, cardiopulmonary bypass; ECMO, extracorporeal membrane oxygenation; IABP, intra-aortic balloon pump.

#### 3.8.3 External validation ROC

**Figure 6:**
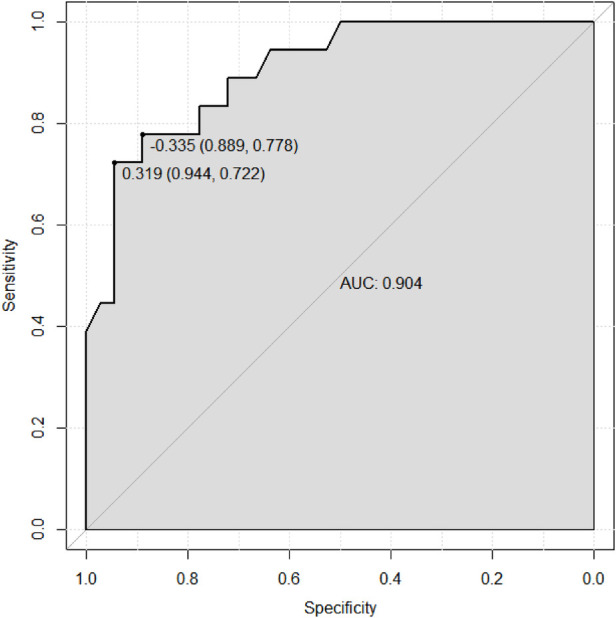
ROC curve of the risk prediction model for HB incidence in the validation group of VHD patients. ROC, receiver operating characteristic; HB, hyperbilirubinemia; VHD, valvular heart disease.

**Figure 7:**
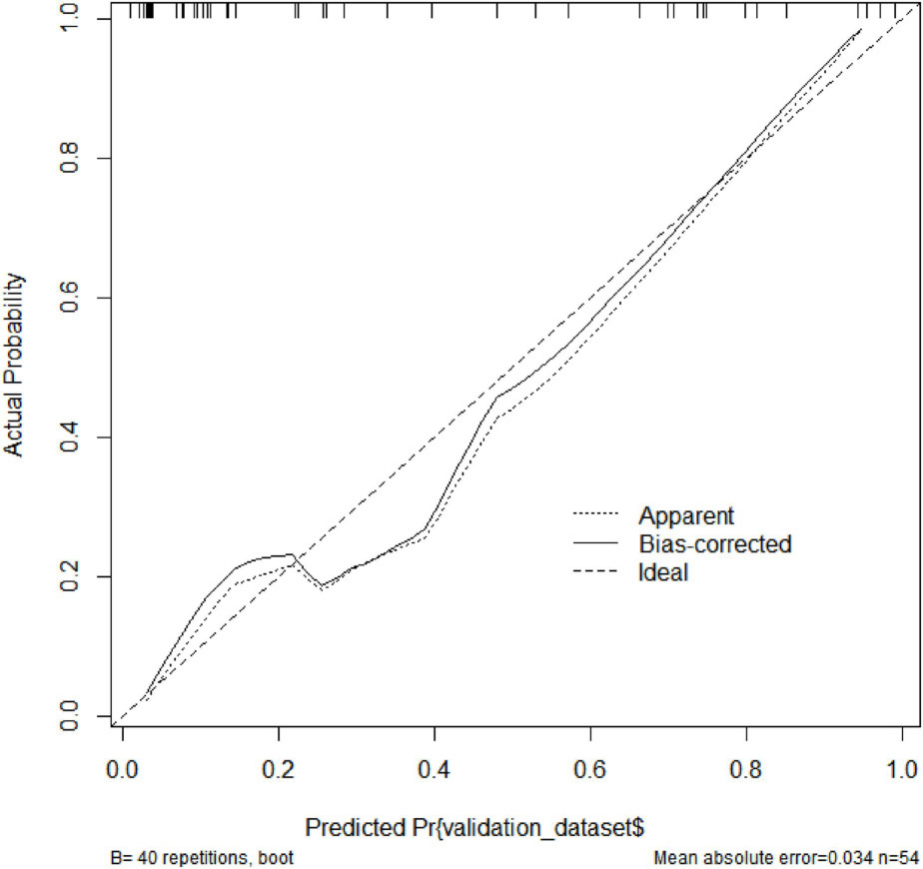
Calibration curve of the risk prediction model in the validation group of VHD patients. VHD, valvular heart disease.

Figures 6, 7 collectively validate the risk prediction model for HB in valvular heart disease patients, demonstrating both its discriminatory power and calibration accuracy. The ROC curve (Figure 6) shows an AUC of 0.904, with a sensitivity of 0.889 and specificity of 0.778 at a threshold of −0.335, indicating excellent ability to distinguish between HB and non-HB patients. The calibration curve (Figure 7) illustrates that the model's predicted probabilities closely match actual outcomes, evidenced by a mean absolute error of 0.034 and close alignment between apparent and bias-corrected curves with the ideal scenario. These findings confirm the model's robustness and reliability in predicting HB incidence, supporting its clinical utility for preoperative risk stratification and personalized intervention planning in VHD patients.

## 4 Discussion

Our study identified several key risk factors for postoperative HB in patients with VHD, including the type of surgery, preoperative MELD score, CPB time, aortic cross-clamp time, total intraoperative blood transfusion volume, and mechanical ventilation time. These findings are significant because they provide insights into the mechanisms behind postoperative HB and offer potential strategies for its prevention and management.

The incidence of HB varied among different types of surgeries, with a higher risk of HB associated with more complex surgical procedures. Patients undergoing aortic valve replacement or tricuspid valve surgery were more likely to develop HB compared to those undergoing mitral valve procedures. This may be attributed to the anatomical and physiological challenges of aortic and tricuspid surgeries, which often require longer CPB and cross-clamp times. Furthermore, multiple valve replacement surgeries, which inherently carry higher complexity and longer perfusion times, are recognized to increase the risk of hyperbilirubinemia. While our study did not include “multiple valve surgery” as a separate categorical variable, its risk profile is captured within our model through the closely correlated variables of “surgery type” and prolonged “CPB time”. For instance, aortic valve surgery involves intricate manipulation of the ascending aorta, potentially increasing the risk of systemic inflammation and microvascular injury (16, 17). Similarly, tricuspid valve procedures are frequently performed in patients with advanced right heart failure, which may compromise hepatic perfusion and exacerbate postoperative liver dysfunction (18, 19). Previous studies have indicated that the number of valve replacements is an independent risk factor for postoperative HB after cardiac surgery (20, 21). But this study emphasizes the role of specific valve types. This distinction could inform preoperative risk stratification, guiding surgeons to prioritize protective strategies for high-risk procedures.

A higher MELD score, reflecting impaired baseline liver function, was strongly linked to HB. The MELD score is a scoring system used to predict the severity and prognosis of liver disease, based on serum bilirubin, serum creatinine, and prothrombin time. A higher score indicates poorer liver function and worse prognosis (22, 23). Research has shown that many patients who develop HB after cardiac surgery often have preoperative liver dysfunction (9). Patients with elevated MELD scores likely have reduced hepatic synthetic capacity and bile excretion efficiency, making them more vulnerable to the metabolic stress of surgery. This aligns with Wang et al., who noted that preoperative liver dysfunction increases the likelihood of postoperative HB due to diminished resilience to surgical trauma (24).

Prolonged CPB and aortic cross-clamp times were independent risk factors for HB. The underlying mechanisms include ischemia-reperfusion injury, systemic inflammation, and hemolysis. During CPB, the liver experiences intermittent hypoperfusion, leading to oxidative stress and impaired bilirubin conjugation. Additionally, the mechanical shear forces of CPB can damage red blood cells, releasing unconjugated bilirubin into circulation. Aortic cross-clamp time further exacerbates this by prolonging myocardial ischemia and increasing the release of inflammatory mediators. These findings are consistent with studies by Pasternack et al. and Wang et al., which indicated that the longer CPB and aortic cross-clamp time, the higher the incidence of postoperative HB (25, 26).

Increased intraoperative blood transfusion volume was a critical predictor of HB. Previous studies have found that increased intraoperative blood transfusion volume may lead to hemolysis, resulting in increased bilirubin load, more severe HB, and delayed peak bilirubin levels. Additionally, transfused red blood cells, particularly when stored for extended periods, are prone to hemolysis, releasing free hemoglobin and bilirubin precursors, leading to increased bilirubin concentration. And its osmotic fragility may be more easily disrupted under the impact of CPB. Once the bilirubin produced during this process exceeds the liver's maximum metabolic capacity, it may lead to bilirubin accumulation, resulting in HB occurrence. Stored blood also contains pro-inflammatory cytokines that may worsen liver injury (27, 28).

The mechanical ventilation time has been found to be positively correlated with the incidence of postoperative HB. This may be because patients with severely compromised respiratory function or extensive surgical trauma require longer mechanical ventilation support after surgery. Prolonged positive pressure ventilation can increase right atrial pressure, inhibit venous return, cause hepatic congestion, further aggravate liver injury, and lead to the occurrence of HB. This mechanism is supported by Lyu et al., who noted that ventilatory support duration correlates with HB severity (29). Additionally, prolonged ventilation may delay recovery of spontaneous breathing, prolonging ICU stays and increasing exposure to nephrotoxic medications, which can synergistically worsen liver function.

Based on the identified risk factors, a predictive model was constructed using nomogram, which is an intuitive data visualization tool that presents the quantities between different categories simultaneously, making it easier to compare the relative quantities between different categories. This helps clinical doctors better understand the dynamic changes in the data (30). Although some variables are intraoperative or postoperative, the model still allows for preliminary risk assessment preoperatively based on surgery type and MELD score, which aids in preoperative communication and resource allocation. The ROC analysis results in this study showed that the nomogram had good accuracy and efficacy in predicting the occurrence of HB in the modeling and validation groups. This indicates that nomogram has good predictive accuracy and can be used by clinicians to assess the risk of postoperative HB in VHD patients based on the aforementioned risk factors, thereby providing individualized and precise interventions to reduce the risk of HB (31).

This model helps identify patients at high risk for postoperative hyperbilirubinemia, enabling intensified liver function monitoring, optimized fluid management, and timely hepatoprotective interventions to improve outcomes. Risk stratification using this model could guide the frequency of postoperative monitoring and intensity of interventions, such as dynamic bilirubin surveillance and early treatment in high-risk patients. The model could be integrated into electronic medical record systems to generate individualized risk scores preoperatively, guiding perioperative liver protection strategies and facilitating precision medicine. The model demonstrates good clinical applicability, assisting clinicians in identifying high-risk patients preoperatively and formulating individualized management plans, which may improve patient outcomes.

While this study confirms known risk factors like CPB time and transfusion volume, it expands the understanding of HB etiology by emphasizing the role of surgical type and MELD score. For example, McSweeney et al. focused on general cardiac surgery but did not differentiate valve-specific risks (11). The current findings suggest that valve type should be considered in risk models for VHD patients. Additionally, the integration of MELD score into predictive modeling offers a practical tool for preoperative counseling and resource allocation. However, the study's retrospective design limits causal inference, and further prospective validation is needed.

This study has several limitations. First, its retrospective, single-center design inherently carries risks of selection bias and unmeasured confounding, such as variations in surgical technique, anesthesia protocols, or specific medication use that could influence liver function. Second, the sample size, while sufficient for initial model development, may limit the generalizability of our findings and the model's stability for predicting very rare outcomes. Third, as noted, we did not explicitly include multiple valve surgery as a variable, and other potentially relevant factors like detailed data on stored blood product age or specific markers of hemolysis were not available. Moreover, we did not differentiate between direct and indirect bilirubin, which could provide further pathophysiological insights into the type of hyperbilirubinemia. Future research should focus on multi-center prospective trials to validate the nomogram in diverse populations. Additionally, mechanistic studies exploring the role of oxidative stress, inflammatory cytokines, and red blood cell metabolism in HB pathogenesis are warranted. Advances in machine learning and wearable monitoring technologies could further refine risk prediction by integrating dynamic physiological parameters.

## 5 Conclusion

In conclusion, the type of surgery, preoperative MELD score, CPB time, aortic cross-clamp time, total intraoperative blood transfusion volume, and mechanical ventilation time are risk factors for postoperative HB in VHD patients. The nomogram constructed based on these risk factors has good predictive value and accuracy. However, this study had limited variables and was retrospective in nature, which has certain limitations. Future studies will include more variables and further validate the constructed predictive model. They also should prioritize translational research to bridge the gap between risk prediction and clinical implementation.

## Data Availability

The original contributions presented in the study are included in the article/Supplementary Material, further inquiries can be directed to the corresponding author.

## References

[1] Ajmone Marsan N Delgado V Shah DJ Pellikka P Bax JJ Treibel T Valvular heart disease: shifting the focus to the myocardium. Eur Heart J. (2023) 44:28–40. 10.1093/eurheartj/ehac504 36167923 PMC9805407

[2] Eleid MF Nkomo VT Pislaru SV Gersh BJ. Valvular heart disease: new concepts in pathophysiology and therapeutic approaches. Annu Rev Med. (2023) 74:155–70. 10.1146/annurev-med-042921-122533 36400067

[3] Kisling A Gallagher R. Valvular heart disease. Prim Care. (2024) 51:95–109. 10.1016/j.pop.2023.08.003 38278576

[4] McCarthy PM Whisenant B Asgar AW Ailawadi G Hermiller J Williams M Percutaneous MitraClip device or surgical mitral valve repair in patients with primary mitral regurgitation who are candidates for surgery: design and rationale of the REPAIR MR trial. J Am Heart Assoc. (2023) 12:e027504. 10.1161/JAHA.122.027504 36752231 PMC10111491

[5] Wang X Fan X Ma Y Zhu L Wang T Liu J Transcatheter mitral valve repair versus transcatheter mitral valve replacement in patients with mitral insufficiency. Arch Med Res. (2023) 54:145–51. 10.1016/j.arcmed.2022.12.009 36642671

[6] Saksena D Choudhary A Varma S Shetty S Jain V. Long-term outcomes of valve replacement with mechanical prosthesis in patients with valvular heart disease: a single-center retrospective study. Cureus. (2025) 17:e84655. 10.7759/cureus.84655 40546510 PMC12182600

[7] Chen X Bai M Zhang W Sun S Chen X. The incidence, risk factors, and prognosis of postoperative hyperbilirubinemia after cardiac surgery: a systematic review and meta-analysis. Ann Palliat Med. (2021) 10:7247–57. 10.21037/apm-21-410 34263619

[8] Chen X Bai M Zhao L Yu Y Yue Y Sun S Time to peak bilirubin concentration and advanced AKI were associated with increased mortality in rheumatic heart valve replacement surgery patients with severe postoperative hyperbilirubinemia: a retrospective cohort study. BMC Cardiovasc Disord. (2021) 21:16. 10.1186/s12872-020-01830-5 33407165 PMC7789141

[9] Hunt M de Jong IEM Wells RG Shah AA Russo P Mahle M Conjugated hyperbilirubinemia is associated with increased morbidity and mortality after neonatal heart surgery. Cardiol Young. (2024) 34:1083–90. 10.1017/S1047951123004158 38105562

[10] Liao P Wang X Dong H Chai D Yue Z Lyu L. Hyperbilirubinemia aggravates renal ischemia reperfusion injury by exacerbating pink1-parkin-mediated mitophagy. Shock. (2023) 60:262–71. 10.1097/SHK.0000000000002160 37278995

[11] McSweeney ME Garwood S Levin J Marino MR Wang SX Kardatzke D Adverse gastrointestinal complications after cardiopulmonary bypass: can outcome be predicted from preoperative risk factors? Anesth Analg. (2004) 98:1610–7. 10.1213/01.ANE.0000113556.40345.2E 15155313

[12] Kwon JY Nietert PJ Rockey DC. Hyperbilirubinemia in hospitalized patients: etiology and outcomes. J Investig Med. (2023) 71:773–81. 10.1177/10815589231180498 37386866 PMC10824167

[13] Raveendran D Penny-Dimri JC Segal R Smith JA Plummer M Liu Z The prognostic significance of postoperative hyperbilirubinemia in cardiac surgery: systematic review and meta-analysis. J Cardiothorac Surg. (2022) 17:129. 10.1186/s13019-022-01870-2 35619178 PMC9137213

[14] Otto CM Nishimura RA Bonow RO Carabello BA Erwin JP 3rd Gentile F 2020 ACC/AHA guideline for the management of patients with valvular heart disease: a report of the American College of Cardiology/American Heart Association joint committee on clinical practice guidelines. J Am Coll Cardiol. (2021) 77:e25–197. 10.1016/j.jacc.2020.11.018 33342586

[15] Sharma P Ananthanarayanan C Vaidhya N Malhotra A Shah K Sharma R. Hyperbilirubinemia after cardiac surgery: an observational study. Asian Cardiovasc Thorac Ann. (2015) 23:1039–43. 10.1177/0218492315607149 26405017

[16] Del Val D Panagides V Mestres CA Miró JM Rodés-Cabau J. Infective endocarditis after transcatheter aortic valve replacement: JACC state-of-the-art review. J Am Coll Cardiol. (2023) 81:394–412. 10.1016/j.jacc.2022.11.028 36697140

[17] Mengi S Januzzi JL Jr. Cavalcante JL Avvedimento M Galhardo A Bernier M Aortic stenosis, heart failure, and aortic valve replacement. JAMA Cardiol. (2024) 9:1159–68. 10.1001/jamacardio.2024.3486 39412797

[18] Hahn RT Makkar R Thourani VH Makar M Sharma RP Haeffele C Transcatheter valve replacement in severe tricuspid regurgitation. N Engl J Med. (2025) 392:115–26. 10.1056/NEJMoa2401918 39475399

[19] Hausleiter J Stolz L Lurz P Rudolph V Hahn R Estévez-Loureiro R Transcatheter tricuspid valve replacement. J Am Coll Cardiol. (2025) 85:265–91. 10.1016/j.jacc.2024.10.071 39580719

[20] Gao Y Li D Dong H Guo Y Peng Y Liu Y Risk factors analysis of hyperbilirubinemia after off-pump coronary artery bypass grafting: a retrospective observational study. J Cardiothorac Surg. (2021) 16:294. 10.1186/s13019-021-01678-6 34629102 PMC8503987

[21] Mahboubi R Kakavand M Soltesz EG Rajeswaran J Blackstone EH Svensson LG The decreasing risk of reoperative aortic valve replacement: implications for valve choice and transcatheter therapy. J Thorac Cardiovasc Surg. (2023) 166:1043–53.e1047. 10.1016/j.jtcvs.2022.02.052 35397951

[22] Hudson D Valentin Cortez FJ León IHD Malhi G Rivas A Afzaal T Advancements in MELD score and its impact on hepatology. Semin Liver Dis. (2025) 45:236–51. 10.1055/a-2464-9543 39515784

[23] Pathare P Elbayomi M Weyand M Griesbach C Harig F. MELD-score for risk stratification in cardiac surgery. Heart Vessels. (2023) 38:1156–63. 10.1007/s00380-023-02262-9 37004541 PMC10372113

[24] Wang Z Chen T Ge M Chen C Lu L Zhang L The risk factors and outcomes of preoperative hepatic dysfunction in patients who receive surgical repair for acute type A aortic dissection. J Thorac Dis. (2021) 13:5638–48. 10.21037/jtd-21-1051 34795914 PMC8575816

[25] Pasternack DM AlQahtani M Zonana Amkie R Sosa LJ Reyes M Sasaki J. Risk factors and outcomes for hyperbilirubinaemia after heart surgery in children. Cardiol Young. (2020) 30:761–8. 10.1017/S1047951120000967 32366349

[26] Wang Z Xu J Cheng X Zhang L Wang D Pan J. Hyperbilirubinemia after surgical repair for acute type a aortic dissection: a propensity score-matched analysis. Front Physiol. (2022) 13:1009007. 10.3389/fphys.2022.1009007 36299262 PMC9589278

[27] Farag M Veres G Szabó G Ruhparwar A Karck M Arif R. Hyperbilirubinaemia after cardiac surgery: the point of no return. ESC Heart Fail. (2019) 6:694–700. 10.1002/ehf2.12447 31095903 PMC6676269

[28] Fernández AL Baluja A Al-Hamwy Z Alvarez J. Postoperative hyperbilirubinemia and Gilbert’s syndrome in patients undergoing cardiac surgery. Ann Card Anaesth. (2019) 22:207–9. 10.4103/aca.ACA_48_18 30971605 PMC6489403

[29] Lyu L Song H Gao G Dong H Liao P Shen Z Impact of hyperbilirubinemia associated acute kidney injury on chronic kidney disease after aortic arch surgery: a retrospective study with follow-up of 1-year. J Cardiothorac Surg. (2022) 17:242. 10.1186/s13019-022-01992-7 36175925 PMC9524110

[30] Li S Zhang H Liao X Yan X Chen L Lin Y The occurrence of early atrial fibrillation after cardiac valve operation and the establishment of a nomogram model. Front Cardiovasc Med. (2023) 10:1036888. 10.3389/fcvm.2023.1036888 37139139 PMC10150109

[31] Lin W Chen B Yang K Kuang L. Risk assessment of hyperbilirubinemia using a three-factor model after cardiac surgery. BMC Surg. (2025) 25:63. 10.1186/s12893-024-02731-6 39948559 PMC11823160

